# Children’s subjective uncertainty-driven sampling behaviour

**DOI:** 10.1098/rsos.231283

**Published:** 2024-04-24

**Authors:** Martina de Eccher, Roger Mundry, Nivedita Mani

**Affiliations:** ^1^ Psychology of Language Department, University of Göttingen, Göttingen 37073, Germany; ^2^ Leibniz Science Campus “Primate Cognition”, Göttingen 37077, Germany; ^3^ Cognitive Ethology Laboratory,German Primate Center, Leibniz Institute for Primate Research, Göttingen 37077, Germany; ^4^ Department for Primate Cognition, Johann-Friedrich-Blumenbach Institute, Georg-August-University Göttingen, Göttingen 37077, Germany

**Keywords:** active learning, sampling, information-seeking behaviour, uncertainty reduction, knowledge gaps, cross-situational word learning

## Abstract

Are children and adults sensitive to gaps in their knowledge, and do they actively elicit information to resolve such knowledge gaps? In a cross-situational word learning task, we asked 5-year-olds, 6- to 9-year-olds and adults to estimate their knowledge of newly learned word–object associations. We then examined whether participants preferentially sampled objects they reported not knowing the label in order to hear their labels again. We also examined whether such uncertainty-driven sampling behaviour led to improved learning. We found that all age groups were sensitive to gaps in their knowledge of the word–object associations, i.e. were more likely to say they had correctly indicated the label of an object when they were correct, relative to when they were incorrect. Furthermore, 6- to 9-year-olds and adults—but not 5-year-olds—were more likely to sample objects whose labels they reported not knowing. In other words, older children and adults displayed sampling behaviour directed at reducing knowledge gaps and uncertainty, while younger children did not. However, participants who displayed more uncertainty-driven sampling behaviour were not more accurate at test. Our findings underscore the role of uncertainty in driving 6- to 9-year-olds’ and adults’ sampling behaviour and speak to the mechanisms underlying previously reported performance boosts in active learning.

## 1. Introduction

Information about entities and events in the world is precious. It allows us to better weigh the options available to us, optimize our interactions with the environment and improve connections with like-minded others. However, information is abundant and incurs a processing bottle-neck because we cannot attend equally to all sources of information available to us.

Research on active learning suggests that learners may cope with this processing bottle-neck by actively shaping their learning experience, choosing what and when they want to learn, from whom they want to receive information or the order and pace at which they receive information (see [1] for a review). Such active elicitation of information has positive effects on learning, with participants showing improved learning and retention of the information they chose to receive relative to passive observational experience ([2–6]; but see [7]).

For example, Kachergis *et al*. [4] presented adults with a cross-situational word learning task, where participants were required to learn the mappings of words to objects. Typically, in cross-situational learning tasks, participants can disambiguate the label–object pairings for some of the objects after a few trials while others may remain ambiguous for longer. Participants were divided into two groups. In the active selection condition, participants could choose to stop seeing pairings they had already disambiguated and focus on learning as yet ambiguous pairings. In the passive selection condition, participants were presented with randomly selected word–object pairings. The results suggested increased accuracy in the active participants’ learning with improved subsequent recognition of the word–object pairings, relative to the participants who were passively presented with randomly selected pairings ([4]; see also [8] for similar findings with children).

Such an active boost in learning is explained by a number of different potential mechanisms. For instance, learners may simply attend more to information they actively solicited, due to their having solicited this information [6]. Active learning may also allow the learner to select information tailored to their current state of knowledge, i.e. information that they are close to learning, otherwise known as the region of proximal learning [6,9]. In other words, active learning allows learners to focus on material that they are uncertain about while avoiding wasting time and resources on information they already possess (cf. [4] above).

The efficacy of the latter mechanism is, however, contingent on learners being aware of what they are and are not uncertain about. In other words, the suggestion that active learning boosts learning due to learners being able to selectively attend to information they are uncertain about only holds true if learners have some implicit or explicit awareness of gaps in their knowledge. There is some evidence in the literature that children are indeed sensitive to uncertainty, although they err on the side of overestimating their knowledge [10,11]. Children further develop their explicit metacognitive abilities, i.e. the ability to judge explicitly whether they know or do not know something [12] through their pre-school years. By around 5 years of age, children seem to be able to accurately estimate their knowledge, i.e. they are more likely to say they answered a question correctly when they did, compared with when they answered incorrectly [13–15].

Information gap theories of sampling [16] suggest that children and adults actively seek information that reduces their uncertainty or fills a gap in their knowledge (see also [17]). Some studies suggest that children’s sampling behaviour may be directed at reducing uncertainty. In a recent study, children and adults were trained on a set of novel word–object associations. The task was manipulated so that some word–object associations could not be disambiguated over the course of the training session, while others could. Participants preferentially sampled objects that remained ambiguous at the end of training [18]. Taken together, this literature suggests that children are able to make relatively accurate judgements about what they know and what they do not know and exhibit exploratory behaviour in situations of uncertainty or missing knowledge.

Furthermore, previous studies with adults suggest that subjective confidence may actually be a better predictor of information seeking than putting participants in objectively uncertain situations in perceptual and decision-making tasks. Thus, adults tended to favour exploration over exploitation in a multi-armed bandit task when their confidence in their value beliefs was lower [19]. Moreover, adults would take the opportunity to view a stimulus again before committing to a decision when they were less confident in their answer [20], and when asked to choose the perceptual decision task in which they performed better, participants’ choices were predicted by the accuracy of the answer and not by the difficulty of the task, suggesting that adults rely on their subjective confidence in decision making [21]. With regard to children, studies find that children’s confidence in the correctness of their response predicted their decision on whether to have the response evaluated by an experimenter [13]. Children also sought help more often in a perceptual identification task when they were more uncertain about their answers [22]. What remains unclear is the role of such subjective awareness of uncertainty in uncertainty-reducing information-seeking behaviour in word learning.

Indeed, previous research on curiosity in word learning investigating sampling behaviour in children and adults has typically manipulated the learning environment to create objective uncertainty, e.g. rendering some of the word–object associations ambiguous [23]. However, these tasks do not consider whether the learners were sensitive to the uncertainty induced by the task. Indeed, an ambiguous environment does not: (i) necessarily imply that participants were subjectively uncertain about the ambiguous mappings presented, and (ii) that being aware of uncertainty was critical to their eliciting information in cases of uncertainty. Thus, for instance, some proposals suggest that children may randomly select one object as the referent of a label in cases of ambiguity [24,25], and may, therefore, not be aware that they are in a situation of uncertainty in the tasks described above. Against this background, the current study examines whether adults and children are sensitive to gaps in their knowledge and whether they actively solicit information about items they previously indicated having a knowledge gap about.

In particular, we trained adults and children on a set of novel word–object associations in the context of a cross-situational word learning task. Similar to Kachergis *et al*. [4], all word–object associations can be disambiguated during the training phase. However, given what we know of children’s performance in such tasks, children were unlikely to learn all the word–object associations presented. Next, we asked participants to judge whether they know the label of each of the presented novel objects, followed by a sampling phase where they could choose a subset of the objects whose label they want to hear again. After this, participants were tested on their knowledge of each of the novel word–object associations. We examined whether participants preferentially sampled uncertain word–object associations, i.e. objects whose label they previously reported not knowing, relative to associations they reported knowing. We hypothesized that both adults and children would be able to accurately estimate whether they know the labels of the objects or not. Furthermore, we expected that participants would be more likely to choose to hear the labels of objects whose label they reported not knowing compared with objects whose labels they reported knowing, as an index of their uncertainty-driven sampling behaviour. Finally, given the proposed benefits of uncertainty-reducing active selection on information retention, we expected participants who sampled more objects whose labels they reported not knowing to be more accurate at test.

## 2. Material and methods

The experiment was designed using PsychoPy (version 2022.1.4 [26]). It was distributed to adult participants via pavlovia.org, who took part in the study online and were instructed to use either a computer or a tablet. Child participants completed the experiment in the lab or in the local museum, on an iPad (screen size: 10.9 or 12.9 inch). In the lab, children sat at a table with the iPad positioned in front of them. In the local museum, children took part in a room with limited traffic at a quiet time, using noise-cancelling headphones. Analyses with adults and 5-year-old children, data exclusion and planned models were pre-registered on the Open Science Framework (adults; 5-year-olds). Data collection for 6- to 9-year-olds followed pre-registered data collection with 5-year-olds, and was not pre-registered.

The study included a further test phase investigating the effects of feedback on learning, whose results will be followed up on in another paper. In brief, we found no effects of feedback in this study and are following this up in subsequent studies. While for the purposes of the current manuscript, the study was an entirely within-subjects design, the study had a between-subject design with regard to the role of feedback on later performance (details provided below).

### 2.1. Participants

We recruited 50 adult participants over 18 years of age (Median = 21, Q1 = 20, Q3 = 25 years, range 18–40 years, 43 females) among university psychology students through a student blog. Participants received credits in exchange for their participation. All participants spoke German as their mother tongue. We recruited 67 children aged 5 years (M = 65.84, s.d. = 3.29 months, range 60–72 months, 37 females) from the institute’s database. Seven participants were excluded prior to data analysis because of technical issues. Following pre-registered data collection for the 5-year-olds, we additionally tested 66 children aged 6–9 years (M = 7.48, s.d. = 0.66 years, range 6–9 years, one child was unable to provide their exact age but was in grade 2) in the local museum. Children received a book in return for their participation in the study. Informed consent for participation in the experiment was obtained from caregivers of children and from adult participants. Participants were predominantly White, reflecting the population of the place where the study was carried out. The study was approved by the Institute’s Ethics Committee.

### 2.2. Stimuli

Each participant was presented with six random objects taken from a set of 15. Objects were images of aliens taken from previous studies [8]. To reduce the complexity of the random effect structure, each participant was presented with a unique set of six novel labels from a total of 360 labels. This way, labels were fully nested in participants and we did not need to include the random effect of label in the analysis models. All labels were German-sounding pseudowords made of two syllables (e.g. kenken, gemu), recorded by a female German native speaker.

### 2.3. Procedure

The study consisted of a knowledge judgement practice phase, a cross-situational word learning phase, a knowledge judgement phase, sampling phase, a test phase and a retest phase. The children’s experiment was presented in the context of the story of Luna, a cat astronaut who introduced the child to her alien friends. In the children’s experiment, instructions to all phases were provided orally (recorded by a female German native speaker), while in the adult experiment, instructions were written on the screen and there was no frame story. The experiments were otherwise identical. All details about the instructions that children and adults received (both translated to English and in their original language, German) and the design of the experiment are included in the electronic supplementary material.

#### 2.3.1. Knowledge judgement practice phase

First, participants practised providing judgements of their state of knowledge. A series of four images was presented on the screen, two of which were likely to be familiar to the children (e.g. tree) and two of which were unfamiliar to the children (e.g. antique household items). Participants were asked to indicate whether they knew the label of the object in each image, by clicking on one of two coloured buttons (red: I do not know the label of this object, green: I know the label of this object). While the function of each button was explained to children participants (‘[…] if you know how the object that you see is called, tap on the green button. If you don’t know how the object that you see is called, tap on the red button.’), an arrow indicated the corresponding button.

#### 2.3.2. Cross-situational word learning phase

**Figure 1.**
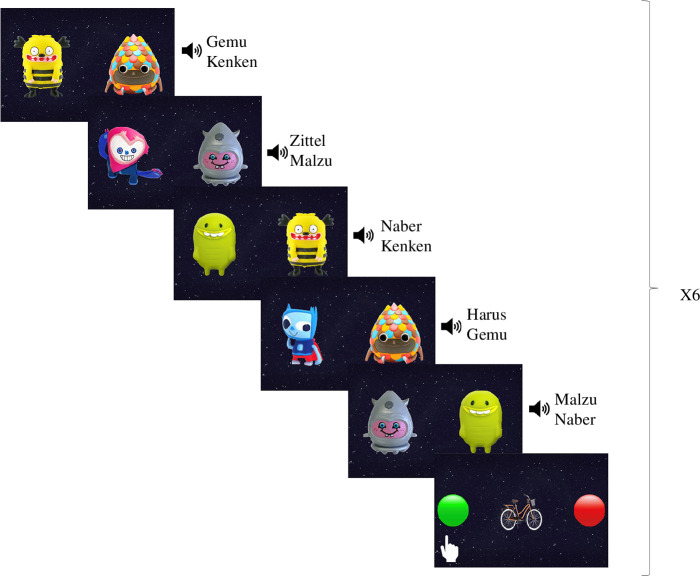
Design of the cross-situational word learning phase. For every five cross-situational learning trials, there was a knowledge judgement-control trial. In total, there were 30 cross-situational word learning trials and six knowledge judgement-control trials.

Participants were then trained on six novel object–label associations. This phase was introduced with the following instructions: ‘Soon you will see Luna’s friends and hear their names. Can you try to remember all their names? It’s very important! Luna will need your help soon to find her friends. Are you ready?’. Across 30 trials, participants saw two objects side-by-side on the screen and heard their labels in random order, e.g. ‘kenken, gemu’ (see figure 1). Within a single trial, the association between the labels and the objects remained ambiguous, i.e. within a trial, it remained unclear which of the two objects was the kenken and which was the gemu. Across trials, however, participants could use the frequency with which particular objects co-occurred with particular labels to infer the intended word–object associations. Every five cross-situational word learning trials, participants were presented with a knowledge judgement-control trial. In each of these trials, participants were asked to tap on one of two buttons to rate whether they knew the label of the novel/familiar object on the screen (see §2.3.4). Note that in this phase, participants were only tested on their knowledge judgement of a separate group of novel and familiar items and not the aliens for whom children were instructed to learn the names.

#### 2.3.3. Knowledge judgement phase

**Figure 2.**
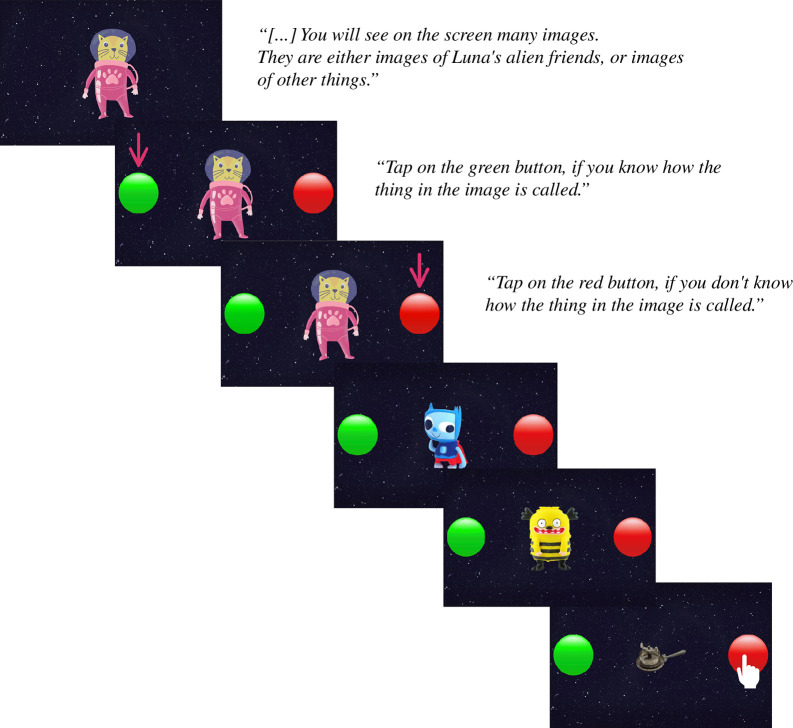
Design and translation of the German instructions presented in the knowledge judgements phase. In this phase, participants rated their knowledge of the labels of the six objects they saw in the cross-situational word learning phase, in addition to three novel or familiar objects for control.

Next, participants were asked to rate whether they knew (or did not know) the label of each of the six novel objects whose name they were taught during the word learning phase. Here, participants saw each of the six objects, one at a time, and were asked to indicate for each object whether they knew the label of the object or not, by clicking on either the green (I know) or the red (I don’t know) button, as in the practice phase (see figure 2).

##### 2.3.3.1. Knowledge judgement-control trials

In a series of control trials, scattered across the cross-situational word learning phase (six trials, see figure 1) and knowledge judgement phase (three trials), participants also rated whether they knew the labels of five familiar objects and four unfamiliar objects. These ratings were used to check whether children understood the rating tasks.

### 2.3.4. Sampling phase

**Figure 3.**
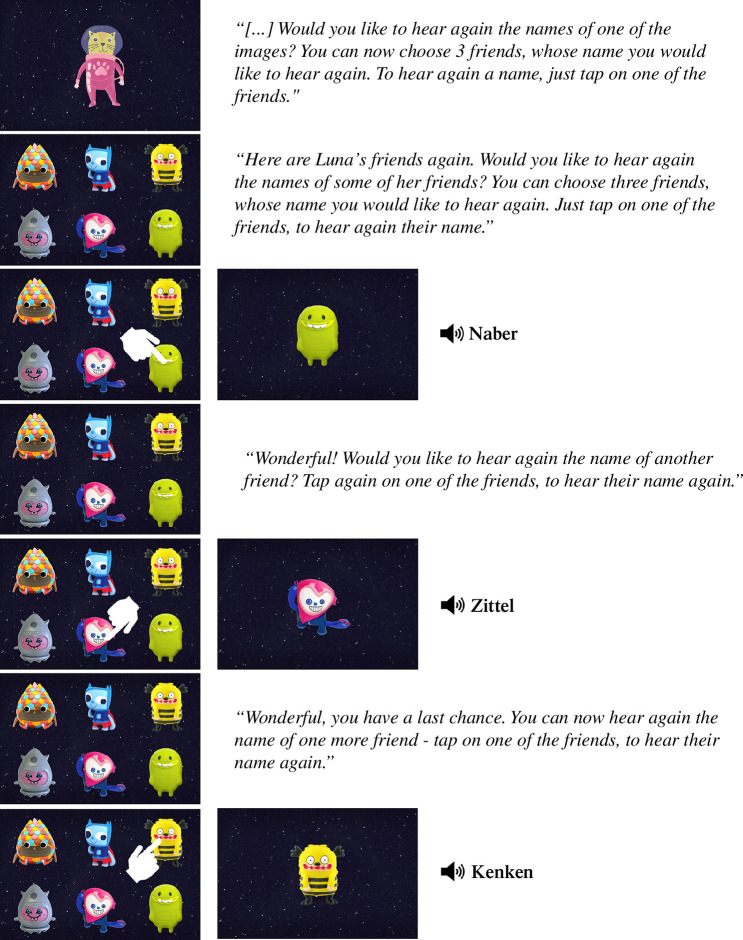
Design and translation of the German instructions of the sampling phase in the children experiment.

Next, participants were allowed to choose three objects across three consecutive trials (i.e. three objects altogether) whose label they wanted to hear again (‘Would you like to hear again the names of some of the images? You can now choose 3 friends, whose name you would like to hear again. To hear a name again, just tap on one of the friends.’). In each sampling trial, all the six objects were presented on the screen simultaneously in a random location across trials, and participants could sample one of the six objects to hear the label of this object in isolation. In each of the three sampling trials, participants selected one of the objects by tapping on it. This object appeared alone in the centre of the screen, while the corresponding label was pronounced (see figure 3). Participants could choose the same object multiple times although they were not explicitly told to do or not to do so. Only one participant in the 6- to 9-year-old group and one in the adult group chose the same object twice, while all the other participants chose three different objects.

### 2.3.5. Test phase

During the test phase, participants were tested on their knowledge of each object–label association in random order, and also asked to rate their confidence in each of their answers. In each test trial, all six objects were presented on the screen in a random location. Participants then heard the label for one of the objects and were asked to click on the object associated with this label ‘[…] Soon she will say a word. Tap on the friend whose name you heard’.

#### 2.3.5.1. Response–confidence trials

After each test trial, participants were asked to rate their confidence in the correctness of their answer by clicking on one of two buttons (‘Are you sure that you answered correctly? Tap on the green button if you are sure that you answered correctly. Tap on the red button if you are not sure that you answered correctly.’).

Following this phase, participants either received or did not receive corrective feedback on their answer and were then tested again on their learning of the word–object associations. We do not report the results of this phase in the current manuscript and are following this up in additional studies.

### 2.4. Planned analysis

All planned models were fitted using the function ‘glmer’ of the package lme4 (version 1.1.30; [27]) on R (version 4.2.0, [28]). For all analyses, three separate models were fitted for the three age groups. All models included a random intercept effect of participant (*n* = 58 levels in models for 5-year-olds; *n* = 64 levels in models for 6- to 9-year-olds; *n* = 50 levels in models with adults) and a random intercept effect of object (*n* = 15 levels in each model for the three age groups). We included random slopes of predictors within random terms when they were theoretically identifiable. Confidence intervals (95%) of model estimates and fitted values were determined by means of a parametric bootstrap (function bootMer of the package lme4; *n* = 1000 bootstraps).

#### 2.4.1. Overall accuracy

To check overall accuracy of participants, we used an intercept-only logistic mixed-effect model. We then compared the intercept with chance level (1/6). For all three age groups, the model was the following:


testaccuracy∼1+(1|participant)+(1|object).

The response *test accuracy* was the correctness of the test answer at each test trial (0 was incorrect, 1 was correct). To test whether the intercept was significantly different from chance level, we determined a *z*-statistic by dividing the difference between 1/6 (in link space) and the estimated intercept by the standard error of the intercept. This indicates how many standard errors the intercept differed from the chance level (1/6 in link space: −1.609). We then determined the associated two-tailed *p*-value using the R function ‘pnorm’.

#### 2.4.2. Accuracy of rating of confidence in the correctness of test responses

To examine whether correct answer at test were more likely to be rated as correct, we used a logistic mixed effects model. The model was specified as follows for 5-year-olds and 6- to 9-year-olds (the model for adults did not include the random slope of test answer within participant as it was not identifiable):


confidencerating∼testaccuracy+(1+testaccuracy|participant)+(1+testaccuracy|object).

The response *confidence rating* indicated the participants’ rating of the correctness of their answer following each test trial (0 was ‘I am not sure my answer was correct’, 1 was ‘I am sure my answer is correct’). The fixed effect predictor *test accuracy* was the correctness of the test answer at each test trial (0 was incorrect, 1 was correct). To test the fixed effect of *test accuracy* and get a more reliable *p*‐value, we compared each model with a model lacking the fixed effect of *test accuracy* but being otherwise identical, using a likelihood ratio test ([29]; R function ‘drop1’).

#### 2.4.3. Preferential sampling of objects whose labels were reported to be unknown

To test whether participants were more likely to sample unknown objects, we fitted the following logistic mixed effect model for 5-year-olds and 6- to 9-year-olds (the model fitted for the adult group also included the random slope of *knowledge judgement* within participant):


$$\begin{array}{l} sampling\;matrix∼knowledge\;judgement+(1\;|\;participant)+(1+knowledge\;\;judgement\;|\;object). \end{array}$$

The response was a two-column matrix, with the number of times each object was sampled (0, 1, 2 or 3) in the first column and the number of times each object was not sampled (out of a total of three times) in the second column (calculated as 3 – number of times the object was chosen [30]). The predictor was participants’ judgements of their knowledge of the label for each particular object after the cross-situational word learning task (0 was ‘I don’t know the label’, 1 was ‘I know the label’).

A problem with this way of setting up the response is that it inflates the sample size. In fact, per participant, the two-column matrix implies that it made 18 decisions although it in fact made only three. As a consequence, one can expect a highly elevated type I error rate. We, therefore, tested the significance of the fixed effect of the knowledge judgement after training by means of a permutation test [31,32]. A permutation test consists of randomizing the data such that datasets are generated for which, by definition, the null hypothesis is true. By creating many such random distributions of the data, one can derive the expected distribution of a test statistic, given the null hypothesis being true. Finally, one can check whether the test statistic of the empirical data is at the edge of this distribution and, thereby, determine significance. Here, we randomized the rows of the two-column matrix representing the response within participants 1000 times, whereby we included the original data as one of those 1000 permutations. As a test statistic, we chose the absolute estimate associated with confidence after training and the *p*-value was finally determined as the proportion of permutations revealing a test statistic at least as large as that of the original data.

#### 2.4.4. Effect of sampling unknown objects on test accuracy

To examine whether participants who sampled more objects whose label they reported not knowing were more accurate at test, we used a logistic mixed effects model testing the effect of the proportion of unknown objects sampled out of the maximum possible choices (three) on accuracy at test,


$$\begin{array}{l} test\;accuracy∼proportion\;of\;unknown\;objects\;sampled+(1\;|\;participant)\;+\;(1+proportion\;of\;unknown\;objects\;sampled\;|\;object). \end{array}$$

The response *test accuracy* was participants’ accuracy at each test trial (0 was incorrect, 1 was correct). The fixed effect predictor was the proportion of objects sampled that participants reported not knowing the label of out of the three total possible sampling choices (*x*/3, *z*-transformed to a mean of zero and a standard deviation of 1).

## 3. Results

A more detailed description of the analysis can be found in the electronic supplementary material. All data and analysis scripts are available at OSF. The final dataset included 50 adults, 58 5-year-olds and 64 6- to 9-year-olds. Two 5-year-olds and two 6- to 9-year-olds were excluded due to poor performance in the knowledge judgement-control trials with familiar and unfamiliar objects, i.e. saying they knew the label of an unfamiliar object or that they didn’t know the label of a familiar object. These children scored less than five of nine points in the knowledge judgement-control trials. On average, children correctly estimated their knowledge of the familiar and unfamiliar objects (5-year-olds: 7.2/9; s.d. = 1.4; 6- to 9-year-olds: 7.5/9, s.d. = 1.3).

### 3.1. Overall accuracy

**Figure 4.**
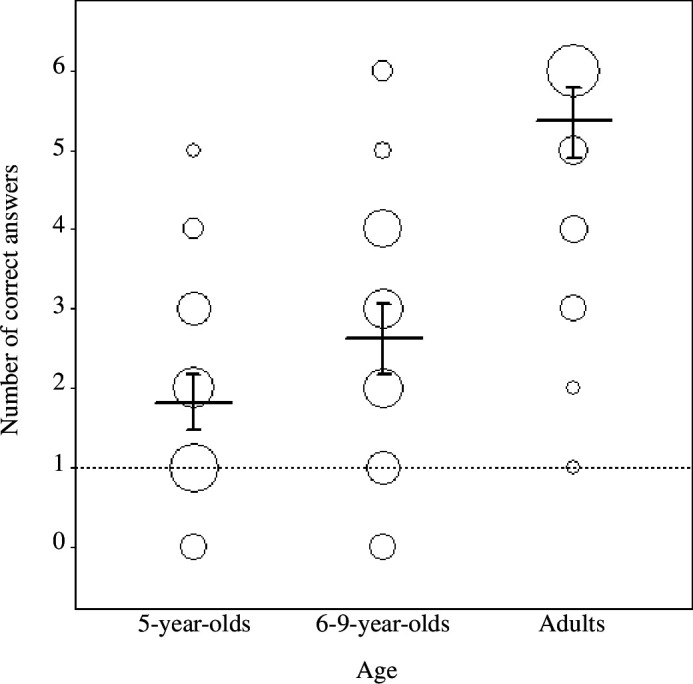
Fitted probability of answering correctly across age groups. Bars indicate 95% confidence intervals. Dots plot empirical data, with the area of the dots proportional to sample size (range: 2–25). The chance expectation for the number of correct answers is 1 (dashed horizontal line).

First, we report the results of the intercept-only model investigating participants’ performance at test (see §2.4.1). Children and adults recognized the word–object associations (5-year-olds: estimate ± s.e. = −0.843 ± 0.134; 6- to 9-year-olds: −0.254 ± 0.154; adults: 2.150 ± 0.402; figure 4, all *p* < 0.001), significantly above chance (−1.609 for randomly choosing the target out of six presented objects). The proportion of trials in which participants responded accurately was 0.31 (s.d. = 0.46) for 5-year-old children, 0.44 (s.d. = 0.50) for 6- to 9-year-old children and 0.81 (s.d. = 40) for adults.

### 3.2. Accuracy of rating of confidence in the correctness of test responses

**Figure 5.**
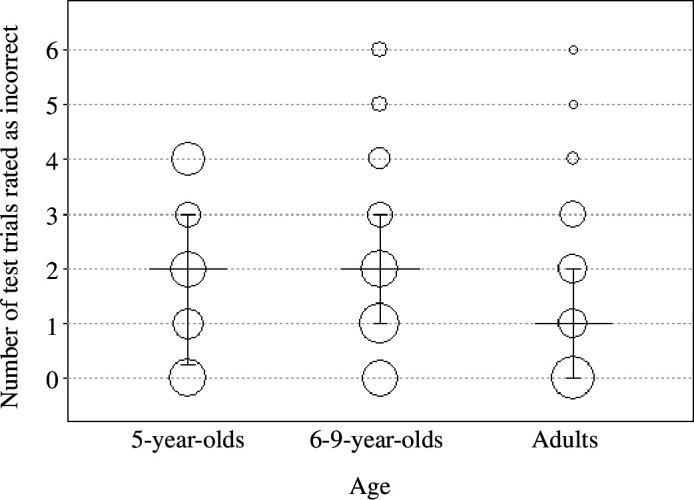
Median number of trials rated as ‘answered incorrectly’ across age groups. Bars indicate interquartile range. Dots plot data points, with the area of the dots proportional to sample size [range: 7–15 (5-year-olds), 3–17 (6- to 9-year-olds) and 1–20 (adults)].

Participants were overall quite confident in the accuracy of their responses (see figure 5). After indicating the intended target at test, participants rated their response as incorrect in 1.85 trials (5-year-olds, s.d. = 1.47), 1.89 trials (6- to 9-year-olds, s.d. = 1.66), or 1.40 trials (adults, s.d. = 1.51).

**Figure 6.**
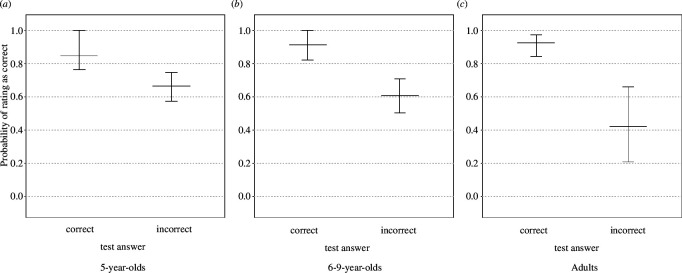
Probability of participants rating their answer as correct when this answer was correct as opposed to when it was incorrect: 5-year-olds (***a***), 6- to 9-year-olds (***b***) and adults (***c***). The segments show fitted probabilities, while the bars indicate 95% confidence intervals.

We examined whether correct test answers were more likely to be rated as correct (see §2.4.2). All age groups were more likely to say they responded correctly when they did respond correctly (fitted probability of saying their response was correct when it was correct: 5-year-olds: 0.85, 6- to 9-year-olds: 0.91, adults: 0.92, compared with when they did not respond correctly: 5-year-olds: 0.66, 6- to 9-year-olds: 0.61, adults: 0.42; 5-year-olds: test answer estimate ± s.e. = −1.043 ± 0.355, *p* = 0.002; 6- to 9-year-olds: −1.888 ± 0.557, *p* < 0.001; adults: −2.808 ± 0.506, *p* < 0.001; figure 6).

### 3.3. Preferential sampling of objects whose labels were reported to be unknown

**Figure 7.**
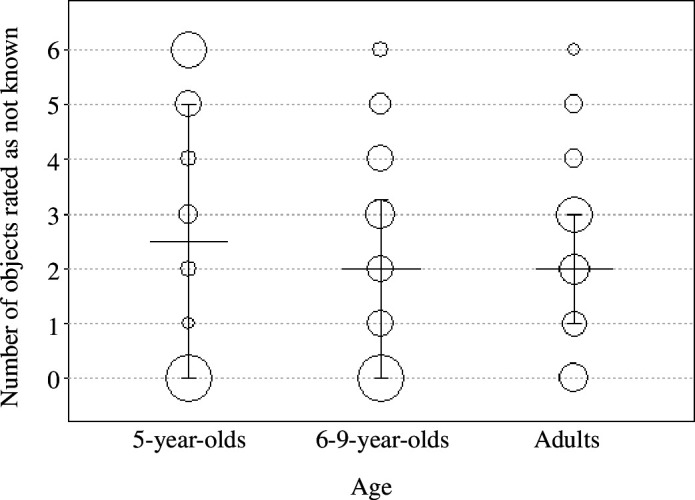
Median number of object–label associations rated as not known after training across age groups. Bars indicate interquartile range. Dots plot data points, with the area of the dots proportional to sample size (range: 2–24 (5-year-olds), 3–23 (6- to 9-year-olds) and 2–14 (adults)).

The mean number of objects rated as ‘not known’ by 5-year-olds was 2.69 (s.d. = 2.59), by 6- to 9-year-olds it was 1.97 (s.d. = 1.93) and by adults it was 2.34 (s.d. = 1.66, see figure 7). Although in principle, participants could sample the same object multiple times, only two participants (one in the 6- to 9-year-old group, one in the adult group) sampled one object twice. All the other participants sampled three different objects.

**Figure 8.**
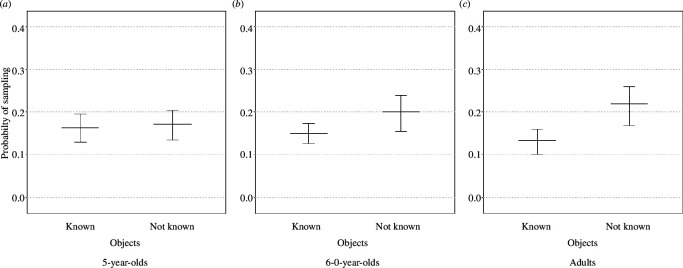
Fitted probability of sampling objects whose labels participants reported not knowing relative to objects whose labels they reported knowing: 5-year-olds (***a***); 6- to 9-year-olds (***b***); adults (***c***).

We report the results of the model investigating preferential sampling of objects whose label was rated as unknown after training (see §2.4.3). Five-year-olds were about equally likely to choose to hear the labels of objects they reported not knowing the label of and objects they previously reported knowing the label of (knowledge judgement estimate ± s.e. = 0.056 ± 0.167, *p* = 0.462]. Six- to nine-year-olds (0.356 ± 0.163, *p* = 0.002) and adults (0.606 ± 0.180, *p* = 0.001), on the other hand, were significantly more likely to choose to hear the labels of objects whose label they previously reported not knowing than of objects whose label they reported knowing (see figure 8).

Post hoc analysis further investigated the preferential sampling of unknown objects. We fitted a linear model with beta error distribution and logit link function. The probability of sampling an unknown object by chance is different for each participant and depends on the number of unknown objects available. To account for this in the model, we calculated the response as follows for each participant:

.$$\frac{Proportion\;of\;unknown\;objects\;sampled\;-\;proportion\;of\;unknown\;objects\;available}{2}+0.5$$

The term *proportion of unknown objects sampled* was calculated as follows: the numerator was the number of unique unknown objects sampled and the denominator was the number of unique objects rated as unknown available, if these were 3 or fewer, and otherwise 3. We gave the denominator an upper limit of 3, to account for the fact that participants could make three sampling choices. As a consequence, if there are four or more unknown objects available, the maximum they can do is to choose three of them. The term *proportion of unknown objects available* was calculated by dividing the number of unique unknown objects available (without upper limit) by 6. To account for the fact that the resulting measure is bound between a value slightly above −1 and a value slightly below 1, we decided to divide the values by 2 and then add 0.5. After this, the response was 0.5 when a participant’s probability of sampling an unknown object was at chance (0 in link space, since logit(0.5) = 0), it was above 0.5 when participants were more likely to sample unknown objects than expected by chance and below 0.5 when participants were more likely to sample known objects. For this analysis, we removed all participants who reported not knowing all or none of the labels of the objects, since these participants necessarily sampled all known or all unknown objects.

Results showed that 5-year-olds (*n* = 20; 38 participants were removed from the analysis) were not more likely than chance to sample unknown objects (intercept estimate ± s.e. = 0.29 ± 0.16; *p* = 0.07); 6- to 9-year-olds (*n* = 38, 26 participants were removed from the analysis) and adults (*n* = 38, 11 participants were removed from the analysis) were more likely than chance to sample unknown objects (6- to 9-year-olds: intercept estimate ± s.e. = 0.55 ± 0.12; *p* < 0.001; adults: intercept estimate ± s.e. = 0.71 ± 0.14; *p* < 0.001).

### 3.4. Effect of sampling unknown objects on test accuracy

We investigated the effect of the proportion of unknown sampled objects on test accuracy (see §2.4.4). We found no evidence that participants who sampled more objects in the sampling phase that they previously reported not knowing the label of, performed more accurately at test (5-year-olds: proportion of unknown objects sampled estimate ± s.e. = 0.025 ± 0.129, *p* = 0.844; 6- to 9-year-olds: −0.143 ± 0.151, *p* = 0.347; adults: −0.596 ± 0.346, *p* = 0.076).

To better account for the different number of objects rated as unknown by different participants, as post hoc analysis, we fitted another logistic mixed effect model. The response was again correctness of test answer, while the predictor was the proportion of unknown objects sampled, with the number of unique unknown objects sampled as numerator and the number of unique unknown objects available as denominator. The fixed effects predictor was z-transformed to a mean of 0 and standard deviation of 1. For this analysis, we removed all participants who had 0 unknown objects, as the proportion of unknown objects cannot be calculated in this way.

Here, 5-year-olds (*n* = 34; 24 participants were removed from the analysis) and 6- to 9-year-olds (*n* = 41, 23 participants were removed from the analysis) were not more likely to be more accurate when they sampled a greater proportion of objects reported to be unknown to them (5-year-olds: proportion of unknown objects sampled estimate ± s.e = 0.31 ± 0.19; *p* = 0.09; 6- to 9-year-olds: proportion of unknown objects sampled estimate ± s.e. = −0.29 ± 0.18; *p* = 0.11). Adults (*n* = 41, nine participants were removed from the analysis) on the other hand, were significantly more likely to be accurate when they sampled a greater proportion of objects whose label they reported not knowing (proportion of unknown objects sampled estimate ± s.e. = = 0.69 ± 0.30; *p* = 0.015).

Further post hoc analysis investigated the impact of sampling on learning, examining whether participants were more accurate on sampled objects, and whether this increased accuracy was greater for unknown objects. We fitted a logistic mixed-effect model with response accuracy at test as the response variable, and the number of times each object was sampled (0, 1, 2 or 3), the knowledge judgement for each object (0 was unknown, 1 was known) and their interaction as fixed-effect predictors.

The interaction between sampling and knowledge judgement was not significant for 5-year-olds (Sampling ∗ knowledge judgement estimate ± s.e. = 0.77 ± 0.55, *p* = 0.16) and 6- to 9-year-olds (Sampling ∗ knowledge judgement estimate ± s.e. = 0.49 ± 0.49, *p* = 0.52). After fitting a null model excluding the term sampling and comparing it with the full model to evaluate the impact of sampling (5-year-olds; *p* = 0.003; 6- to 9-year-olds: *p* = 0.04), we fitted a reduced model excluding the interaction term. This model found that sampling had a significant effect on test accuracy (5-year-olds: sampling estimate ± s.e. = 0.94 ± 0.31, *p* = 0.002; 6- to 9-year-olds: sampling estimate ± s.e. = 0.55 ± 0.22, *p* = 0.015), indicating that 5-year-olds and 6- to 9-year-olds learned from sampling, but did not learn more from sampling when the objects they sampled were judged as unknown.

For adults, the effect of the interaction between sampling and knowledge judgement was significant (Sampling ∗ knowledge judgement estimate ± s.e. = −21.40 ± 7.36, *p* < 0.001). Fitted probabilities suggest that adults’ probability of being correct with their test answer when the corresponding object was rated as known was essentially 1, regardless of whether this object had been sampled or not. In contrast, adults’ probability of being correct with their test answer when the corresponding object was rated as unknown increased when this object was sampled (predicted probability of being correct on a test answer on an unknown object: 0.82 when this was not sampled, 0.83 when this was sampled one time, 0.84 when this was sampled two times).

## 4. Discussion

The current study investigated whether 5-year-olds, 6- to 9-year-olds and adults were sensitive to gaps in their knowledge of newly learned object–label associations and whether such sensitivity drove them to actively solicit information that will reduce these gaps. In what follows, we briefly outline the main results and discuss the implications of our findings.

While all participants showed evidence of having learned the object–label associations, accuracy increased with age, with adults performing almost at ceiling. Furthermore, while 5-year-olds were equally likely to sample objects whose label they reported knowing and not knowing, 6- to 9-year-olds and adults were more likely to sample objects whose labels they reported not knowing. In other words, older children and adults selectively sampled items that reduced the gap in their knowledge of the object–label associations, whereas younger children did not. These findings provide evidence that subjective confidence predicts sampling behaviour in older children and adults during word learning. This aligns with existing literature suggesting that objective uncertainty in the environment guides information-seeking behaviour during word learning [18,33]. Moreover, these findings extend the understanding of the role of subjective confidence in guiding both adults’ [19–21] and children’s [13,22] information-seeking and decision-making behaviour within the context of word learning. Thus, knowledge of one’s own uncertainty in the label of an object leads to older children and adults choosing to elicit the label of this object more than the label of other more familiar objects. This finding, therefore, clarifies the mechanisms underlying children’s sampling behaviour and the critical role that subjective awareness of uncertainty plays in uncertainty-driven sampling behaviour.

Why did younger children not preferentially sample uncertain objects? Previous studies suggest that children’s decision-making behaviour based on subjective uncertainty may emerge at different ages depending on the nature of the task. Thus, while some studies suggest that children exhibit uncertainty-guided decision-making behaviour already from 3 years of age in a perceptual identification task [22], similar uncertainty-driven behaviour is only documented from around 4 years of age in a memory task [13]. These findings may be taken to suggest age-related differences in how subjective uncertainty directs children’s information-seeking behaviour depending on the nature of the representation that children have to judge their (un)certainty about.

Indeed, the ability to reflect on language may be particularly challenging for children [34,35]. Thus, reflecting on one’s own uncertainty in the knowledge of word–object associations and using such judgements to strategically select uncertain objects to increase one’s knowledge might be more difficult for young children—at least relative to simpler perceptual or memory tasks. Indeed, recent work suggests that while children show more elevated body posture, indexing increased positive affect, after a familiar word-recognition task relative to a novel word-recognition task, there was no association between children’s performance in the novel word-recognition task and their posture [36]. In other words, individual differences in how successful children were in the word-recognition task did not explain differences in their positive affect after completing the task. This stands in contrast to the literature finding robust associations between children’s success at a task, e.g. achieving a desired goal or helping someone achieve their goal, and their positive affect [37], which the authors attribute to children’s difficulty in judging their performance in a word learning task.

On the other hand, previous studies suggest that children have quite sophisticated metalinguistic skills and already at 2 years of age, appear to estimate their linguistic confidence, i.e. their looking behaviour is different when they are confident they know the meaning of a word compared with when they are not [38]. General metacognitive abilities develop further during pre-school years. Already at age 3, children can report their confidence in their knowledge accurately [14]. By age 5, children display awareness and are able to explicitly indicate, what they know and what they do not know [12,15]. Indeed, our findings similarly document that all age groups were able to estimate the accuracy of their responses, i.e. they were more likely to indicate that they had answered correctly at test when they had answered correctly, relative to when they had not. Nevertheless, children—and indeed, adults—seemed to also overestimate their knowledge, given that their fitted probability of saying they answered correctly when they answered incorrectly was high (see figure 6). With regard to children, this finding is in line with the literature suggesting that children overestimate their knowledge in absolute terms [15]. Thus, our findings together with the literature to date suggest that while children and adults may have overestimated their knowledge, they displayed sensitivity to gaps in their knowledge of the object–label associations presented.

Despite this, younger children showed no evidence of recruiting such sensitivity in eliciting information that would close their knowledge gaps. Previous studies reporting that (subjective) uncertainty guides decision making in young children tested children on their ability to retrieve the required information before children had the opportunity to display an uncertainty-directed behaviour [10,13,22]. In contrast, in our study, children were not tested on their knowledge before the sampling phase. Younger children may need to try to retrieve information in order to put in place a behaviour that would strategically be directed at filling knowledge gaps. In fact, children rely on retrieval fluency to base their confidence judgements [39]. In other words, by trying to answer a question, one can see how easy it was to access the information and therefore have a clearer idea of where the knowledge gaps are. Introducing a knowledge test before the sampling phase may, consequently, prompt children to actively seek information to fill these gaps. Ongoing studies in our laboratory are currently examining this possibility in more detail.

Moreover, our knowledge judgements were binary, i.e. requiring participants to simply indicate whether they knew a word–object association or not. However, uncertainty is a continuum, and young children appear to be able to discriminate between the extremes of this continuum, i.e. discriminate something that is very uncertain from something that is very certain [40]. For example, in the study of Zettersten & Saffran [18], children sampled ambiguous objects only once ambiguity was made very salient. It might be that 5-year-olds, for whom the task was particularly difficult (on average, they answered correctly in about 30% of the test trials, after sampling), had different levels of uncertainty on their knowledge of the labels for objects. A measure of uncertainty that would allow for more nuanced report of one’s knowledge might have uncovered behaviour directed at reducing this uncertainty.

A further explanation for the findings reported here may be differences in the weighting of sampling strategies across early development. In particular, studies find that younger children display increased random exploratory behaviour, with random exploratory behaviour decreasing between 4 and 9 years of age. For instance, in tasks where they can explore the environment to obtain rewards, younger children continue exploring the reward space in a random manner, even when they have previously identified high-reward locations ([41]; see also [42]). The differences in sampling behaviour reported in the current study are in line with these findings, suggesting that only the older children—and adults—showed evidence of directed uncertainty-driven sampling behaviour, i.e. by selectively sampling objects whose labels they previously reported not knowing, while younger children did not. However, we note that our findings cannot adequately speak to the ontogeny of uncertainty-driven sampling strategies in early development, given the samples tested in the different ages (5-year-olds relative to 6- to 9-year-olds) and future studies should include more balanced samples of children in this age range to strengthen the evidence on the developmental trajectory of uncertainty-driven sampling behaviour.

Finally, our findings speak to the literature on the benefits of active learning, with such uncertainty-reducing information-seeking behaviour allowing learners to shape their learning experience around gaps in their knowledge [6,9]. Such suggestions have been used to explain why there may be a performance boost in active learning, relative to passive learning. However, we found no clear evidence that participants who displayed greater uncertainty-driven sampling behaviour performed better at test. In adults, the lack of an active boost found in the pre-registered analysis could be explained by the fact that adult performance was almost at ceiling. Indeed, post hoc analysis removing all participants who reported knowing all the labels suggested that adults did increase their accuracy when they sampled strategically, i.e. when they sampled a greater proportion of objects whose label they reported not knowing. This post hoc analysis would, therefore, be more in keeping with the mechanisms suggested to explain the active boost in learning at least in adults, i.e. that active elicitation of information may allow participants to target information that they are currently lacking. In both children groups, on the other hand, both the pre-registered and the post hoc analyses lead to similar results, i.e. they indicated a surprising lack of an uncertainty-driven active boost. Given that children’s performance at test was not at ceiling, they could have benefited from targeted uncertainty-driven sampling behaviour, but appear not to have. Other post hoc analysis further suggested that children of both age groups did indeed learn from sampling, i.e. they were more accurate at identifying the objects at test, when they had sampled the label of this object relative to objects they had not sampled. However, this difference in accuracy between sampled and not sampled objects was not greater for unknown objects. While we cannot advance an explanation for this finding, we note that it may be related to previous studies reporting a lack of an active benefit in learning, especially in young children [7,43].

## 5. Conclusion

Taken together, our results underscore the role of uncertainty in driving children and adults’ sampling behaviour. In particular, we found that older children and adults are more likely to sample information they previously indicated not knowing relative to information they previously indicated knowing. Nevertheless, our findings question the mechanisms underlying potential boosts in performance previously attributed to such uncertainty-driven sampling behaviour given that we found no clear evidence that children who displayed greater uncertainty-driven sampling behaviour performed better at test. This highlights the need for further research on the mechanisms underlying previously reported performance boosts in children active learning.

## Data Availability

The data and materials that support the findings of this study are publicly available at OSF [44]. Electronic supplementary material is available online [45].
